# Effects of an aquatic physical exercise program on glycemic control and perinatal outcomes of gestational diabetes: study protocol for a randomized controlled trial

**DOI:** 10.1186/1745-6215-14-390

**Published:** 2013-11-19

**Authors:** José Roberto da Silva, Paulo Sérgio Borges, Karine F Agra, Isabelle Albuquerque Pontes, João Guilherme Bezerra Alves

**Affiliations:** 1 Mother and Child Department, Instituto de Medicina Integral Prof. Fernando Figueira (IMIP), Recife, Brazil

**Keywords:** Aquatic physical exercise, Gestational diabetes mellitus

## Abstract

**Background:**

Gestational diabetes mellitus (GDM) is increasing worldwide and has been associated with adverse perinatal outcomes and high risk for chronic disease both for the mother and for the child. Physical exercise is feasible for diabetic pregnant women and contributes to better glycemic control and to a decrease in adverse perinatal outcomes. However, there are no randomized controlled trials (RCT) assessing the effects of aquatic physical exercise on GDM control and adverse maternal and fetal outcomes.

**Methods/Design:**

An RCT will be conducted at Instituto de Medicina Integral Prof Fernando Figueira (IMIP), Brazil. A total of 72 pregnant women will be studied; 36 gestational diabetics will undergo an aquatic physical exercise program in a thermal pool, 3 times per week over 2 months. The primary endpoint will be glucose level control and use of insulin; secondary endpoints will be the following maternal and fetal outcomes: weight gain during pregnancy, blood pressure, pre-eclampsia diagnosis, intrauterus growth restriction, preterm birth, Cesarean section, macrosomia and maternal or neonatal intensive care admission. Endpoints between intervention and control group will analyzed by t test for unpaired data and χ^2^ test, and the level of significance will set at <0.05.

**Discussion:**

The physical proprieties of water make aquatic exercises ideal for pregnant women. An aquatic physical exercise program developed for GDM women will be trialed in a thermal pool and under the supervision of physiotherapist to ensure compliance. It is expected that this study will provide evidence as to the effect of aquatic physical exercise on GDM control.

**Trial registration:**

ClinicalTrial.gov, NCT01940003.

## Background

The prevalence of gestational diabetes mellitus (GDM), defined as glucose intolerance first diagnosed during pregnancy, is increasing worldwide concurrent with the rise in obesity and type 2 diabetes [1]. According to the International Association of Diabetes and Pregnancy Study Groups (IADPSG) and based on the new screening criteria for GDM from the Hyperglycemia and Adverse Pregnancy Outcomes (HAPO) study, the incidence of GDM has reached almost 18% [2]. GDM has been associated with significant rates of adverse perinatal outcomes [3]. Both GDM women and their offspring also have a high risk of developing obesity, type 2 diabetes and metabolic disease throughout life [4].

Physical activity improves glucose utilization by increasing insulin sensitivity [5]. Physical activity can delay the progression of impaired glucose tolerance in type 2 patients and also contributes to overweight/obesity control, reduction of medication dose and cardiovascular risk factors [6]. Physical exercise is safe for pregnant women and has been recommended at a level of 30 minutes or more on most days of the week, as a helpful adjunctive therapy for GDM [7,8]. Physical activity during pregnancy may contribute to improved levels of maternal glucose tolerance and help prevent GDM [9]. However, a Cochrane systematic review concluded that there was insufficient evidence to recommend, or advise against, diabetic pregnant women enrolling in exercise programs [10].

The physical properties of water make aquatic exercise ideal and safe for pregnant women [11]. Aquatic exercise has been recommended during pregnancy due to it having less impact on articulation, and its ability to increase amniotic fluid diuresis and decrease edema, blood pressure and back pain [12,13]. Furthermore, aquatic exercise during pregnancy may decrease maternal stress, discomfort and improve health-promoting behaviors [14,15]. However, there is insufficient evidence on aquatic exercise due to poor methodological and reporting quality and heterogeneity of non-randomized clinical trials [10,16]. Specifically in relation to GDM, there are no clinical trials testing aquatic exercise as an adjunctive treatment.

### Hypothesis

Aquatic physical exercise is feasible for diabetic pregnant women and contributes to better glycemic control and decreases adverse perinatal outcomes.

### Research goals

To verify the efficacy of an aquatic physical exercise program on GDM control and adverse maternal and fetal outcomes.

## Methods/Design

### Setting and recruitment

The study will take place at Instituto de Medicina Integral Prof. Fernando Figueira (IMIP), Brazil. IMIP is a reference hospital in the Northeast Brazil for mother and child care, and performs about 6,000 deliveries per year. Recruitment will focus on GDM women diagnosed at IMIP or referred to the center for treatment; this is about 50 GDM women per month.

### Participant selection

Obstetrical staff will identify pregnant women recently diagnosed with GDM; following IMIP guidelines, a 75 g oral glucose tolerance test (OGTT) is routinely performed between the 24th and 28th gestational week. These patients will be approached by a member of the study team and permission will be sought after an explanation of the study goals. GDM women will be considered eligible for enrollment if they fulfill all the inclusion criteria and none of the exclusion criteria. Interested patients will be invited to sign a written informed consent.

### Study design

This is an RCT to assess the effects of a 2-month water exercise program on GDM control and perinatal outcomes.

### Inclusion criteria

GDM diagnosis will be based on the IADPSG HAPO study (2 h, 75 g OGTT: fasting glucose ≥92 mg/dl or a 1-h result of ≥180 mg/dl, or a 2-h result of ≥153 mg/dl) [17]. Subjects must be aged 18 to 35 years and physically inactive (<150 minutes per week based on International Physical Activity Questionnaire) [18].

### Exclusion criteria

These are: multiple pregnancy, cervical incompetence, placental dysfunction, intrauterus growth restriction, preterm labor, suspicion of fetal suffering, heart or pulmonary insufficiency and blood hypertension.

### Sample size

The sample size was calculated with the aim of reducing glucose levels by 20% on average in the intervention group. A power of 80% and a level of significance of 5% was accepted, and thus the calculated sample size in each arm was 30 patients. Assuming a dropout of 20%, 72 pregnant women will be included in the study.

### Primary endpoint

Glucose control will be considered as fasting capillary glucose levels of 95 mg/dl or less and postprandial levels of 120 mg/dl according to American Diabetes Association (ADA) guidelines [8]. The mean value of capillary glucose levels (fasting and postprandial) and the number of measurements above recommended values (ADA) will be compared between the groups.

### Secondary endpoints

Weight gain during pregnancy (weight measured at the end of pregnancy minus prepregnancy weight, as reported by the subject); systolic and diastolic blood pressure; pre-eclampsia diagnosis (systolic blood pressure ≥140 mmHg or diastolic blood pressure ≥90 mmHg occurring after 20 weeks of gestation and simultaneous proteinuria of ≥300 mg/24 hours); urinary tract infection; vaginal infections; intrauterus growth restriction (fetal ultrasound parameters) [18]; preterm birth (before 37th gestational week); Cesarean section; birth injury; macrosomia (birth weight above the 90th centile); maternal or neonatal intensive care admission.

### Pre-randomization evaluation

All diabetic pregnant women will have their clinical history recorded and will undergo a complete physical examination to identify diseases that do not allow for the use of the pool or for performing physical exercise.

### Randomization

Randomization will be performed according to a computed-generated allocation (http://www.randomized.com). Pregnant women will be assigned, in a 1:1 ratio, to exercise intervention or usual care. Blinding of the study to the randomization arm is not possible due to the nature of intervention.

### Post-randomization evaluations

To ensure that similar guidelines for GDM clinical treatment are maintained for the two groups, IMIP obstetrical staff will undertake the ongoing GDM management of all trial participants for the period of the study. Participants are usually clinically evaluated at a minimum of every 2 weeks depending on GDM control, according to the IMIP guidelines for GDM. A capillary glucose test is performed at each clinical visit.

Self-monitoring of fingertip capillary blood glucose will be performed after overnight fasting, 2 h after breakfast, 2 h after lunch, and 2 h after dinner. The capillary glycemic profile will be determined every other week until 34 weeks of gestation, and weekly thereafter. All women will receive instructions to measure capillary glycemia with an Accu-Chek Advantage glucose meter (Roche Diagnostics, Indianapolis, IN, USA) and glucose blood strips. Insulin will be introduced when more than two glucose measurements are above the recommended value.

The mean capillary glucose profile will be analyzed post intervention (minimum of five determinations per woman). Insulin or oral hypoglycemia will be compared between the two groups.

All pregnant women will wear a pedometer (Yamax Digi Walker SW-200, Tokyo, Japan) during the whole study. Pedometer readings will provide a measure of physical activity to compare both groups.

### Usual care

Standard dietary and exercise advice will be emphasized before and after randomization for all women. At randomization and each clinical visit, a dietitian and a nurse will provide food intake education and information about GDM treatment.

### Aquatic exercise intervention

Participants commence the exercise session by performing stretching and an active warm-up of 5 minutes duration. Aquatic sessions include walking, walking backwards, swimming laps, jogging, step climbing and strength exercises in the water. After 35 minutes, the participants initiate a 5-minute cool down period.

Pool-based exercise classes will be completed in groups of four to six participants under the instruction of a physiotherapist. The exercise program will be conducted three times per week with each session lasting 45 minutes. This will be conducted from GDM diagnosis (26th to 28th gestational week) to the end of the third trimester (38th to 39th gestational week). Thus, an average of 30 training sessions will be planned for each pregnant woman.

The entire program will be developed in the thermal pool at IMIP; water temperature around 28°C.

All participants will use a heart rate monitor (Polar Electro OY) during the training sessions to ensure that heart rate is consistently under 70% of their age-predicted values.

**Figure 1:**
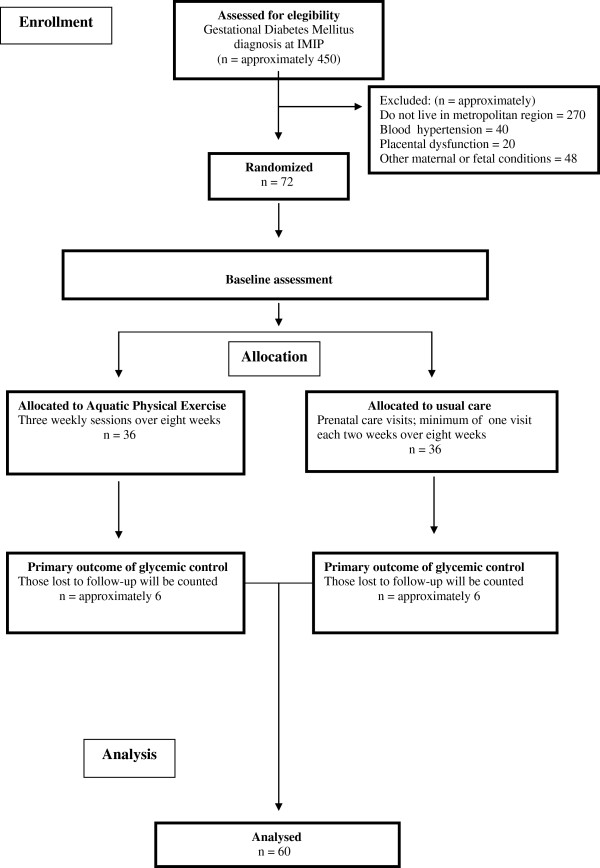
Flow diagram of the aquatic physical exercise trial design.

A summary of the study design is presented in Figure 1.

### Analyses

Maternal and fetal characteristics of the study sample will be presented by group, intervention and control in terms of mean and SD. For group comparisons of glucose levels and perinatal variables, continuous and nominal data will be analyzed by t test for unpaired data and χ^2^ tests, respectively. Data will be analyzed using the intention-to-treat principle. Statistical analysis will be performed with STATA version 3.1 and the level of significance will set to <0.05.

### Ethics

This study was approved by the IMIP Research Ethics Committee. All participants must sign an informed consent before admission to the study. A steering committee was established to monitor the trial.

## Discussion

Currently, there is a lack of evidence regarding the association between physical exercise and GDM control. To the best of our knowledge, this is the first randomized controlled trial evaluating the effects of an aquatic physical exercise program on GDM outcomes. Water provides resistance to muscle movement, resulting in higher workout intensities without gravitational stress and pain as well as a massaging effect. These effects may facilitate the practice of physical exercise by pregnant women and thus increase compliance.

During pregnancy, women decrease their physical activity level, especially in the last trimester. Physical exercise in water is more feasible and may be more suitable for pregnant women. Additionally, doing exercise in groups can contribute to socialization and help ensure greater adherence.

Although our study will be performed in tropical weather, temperature approximately 28°C to 30°C year round, by using a thermal pool our results can be extrapolated to other regions with colder temperatures.

This project has several methodological strengths. First, it is safe for pregnant women. Aquatic physical exercise will be supervised by a physiotherapist, all participants will wear a heart rate monitor and the intensity of the physical exercise will be low to moderate. Only women who live in the metropolitan region will be included and all transportation cost will be covered. Bathing suits and lunch will also be supplied to the participants. We believe that these measures will ensure greater adherence to the study.

Our study has some methodological weaknesses that will be taken into account, but that are unlikely to influence ours findings. Our close monitoring of physical activity among all participants via the wearing of a pedometer may allow us to verify if the two groups are maintaining the same physical activity level outside of the aquatic physical exercise.

In conclusion, this study is expected to provide findings that may reveal the true effect of aquatic physical exercise on GDM control.

## Trial status

This trial is registered under: NCT01940003. Recruitment commenced in August 2013 and is expected to continue until March 2014. Open for recruitment.

## Abbreviations

GDM: gestational diabetes mellitus; HAPO: Hyperglycemia and adverse pregnancy outcomes; IADPSG: International association of the diabetes in pregnancy study group; IMIP: Instituto de Medicina Integral Prof. Fernando Figueira.

## Competing interests

The authors declare that they have no competing interests.

## Authors’ contributions

JGBA is the principle investigator of the study and takes full responsibility for the integrity and the accuracy of the data. Study concept and design: JGBA and JRdSJr. Acquisition of data: JRdSJr, PSB. KFA and IAP. Analysis and interpretation of data: JGBA, JRdSJr, KFA, IAP and PSB. Drafting of the manuscript: JGBA, JRdSJr, KFA, IAP and PB. Critical revision of the manuscript for important intellectual content: JGBA and JRdSJr. Funding obtaining: JGBA. Administrative, technical, or material support: JGBA and JRdSJr. Supervision: JGBA. All authors read and approved the final manuscript.
